# Perspectives on learning to practice reflexivity while engaging communities in implementation science

**DOI:** 10.3389/frhs.2022.1070444

**Published:** 2023-01-16

**Authors:** Eva N. Woodward, Irenia A. Ball

**Affiliations:** ^1^ Department of Psychiatry, University of Arkansas for Medical Sciences, Little Rock, AR, United States; ^2^ VA Center for Mental Healthcare and Outcomes Research, Central Arkansas Veterans Healthcare System, North Little Rock, AR, United States

**Keywords:** implementation practice, implementation science, community engagement, reflexivity, community-based participatory research, equity, service users, consumer

## Abstract

**Background:**

As implementation scientists and practitioners engage community members and service users, reflexivity rises as a critical approach for managing power imbalances and effective collaborative work to promote equity. Reflexivity is an approach for acknowledging scientists’ own positions, including their understanding and limits of how they view their phenomena of inquiry. We describe our perspective practicing reflexivity as an implementation science team new to community engagement.

**Methods:**

We spent over two years learning principles of Community-Based Participatory Research (CBPR) to inform implementation science and practice, then applied CPBR principles into a new community-academic partnership in August 2020 for and with veterans of the United States Military living in rural Arkansas. We used five methods to practice reflexivity for the first time: identifying positionality, writing fieldnotes, obtaining mentorship on technical aspects, comparing head notes, and consulting reference materials.

**Discussion:**

We found multiple methods for practicing reflexivity to be feasible, although difficult to stay consistent with busy schedules. Fieldnotes especially required commitment and were important not to minimize. Written fieldnotes enabled us to reflect on successes and missteps, funneling into action planning. Head notes allowed emotional catharsis and to generate insights based on each other's perspectives. Referencing books or course modules reminded us of ideal CBPR principles. Discussion with mentors helped us with technical aspects and balancing real-world challenges with ideal CBPR principles. Our methods to practice reflexivity were valuable and directly impacted process and research outcomes. Future training for implementation science and practice might consider reflexivity practice as a core competency.

## Introduction

As implementation scientists and practitioners engage community members and service users in their work, reflexivity rises to the forefront as a critical approach for managing power dynamics and collaborative effective work to promote equity. Practicing reflexivity is an approach for acknowledging scientists' own positions, including their understanding and limits of how they view their phenomena of inquiry. Thus, practicing reflexivity (or not) affects research and knowledge translation. However, foregrounding our work in reflexivity is not a clear objective of training for implementation scientists or practitioners, and is not, in our experience, common practice. We describe our experience practicing reflexivity as an implementation science team new to community engagement. One first step in practicing reflexivity is to examine the researcher's positionality – their own identity and lived experiences, and how they manifest in more power or greater risk of marginalization.

### Eva woodward

As an implementation scientist focused on improving health equity by reducing disparities in healthcare, I was a novice in community-engaged research in 2020. Community-engaged research is a gold standard for health disparities research (1), so it seemed important in my research program. My focus on health equity stems from my own poor health and healthcare due to my experience growing up low-income, in a rural area of the United States (U.S.), underserved by healthcare providers, with a single father, and being first in my family to attend college. I identify as a woman, disadvantaged by patriarchal behaviors and systems. My racial identity is White, I am not Hispanic or Latina, and I am a cisgender, heterosexual person, thus benefitting from, and likely enacting, behaviors and racist systems favoring whiteness. Now, I am a clinical health psychologist who holds a doctorate and earns over double the median income in my Southern United States city and more than anyone in my family ever earned. I am not a military veteran and have worked across three Veterans Health Administration sites for ten years.

### Irenia ball

As a research assistant working with an implementation scientist, I provide aid to principal investigators and participants to carry out research. Although my role is not to oversee studies, I must understand the accepted social norms and health disparities the community endure and my own social statuses. I am a college-educated Black woman with a sociology degree who has lived in urban areas. The importance of health equity was established when my father worked in school-based health clinics in New Orleans and I witnessed adversities people endured such as poverty, unemployment, poor healthcare, and inconsistent transportation. Through this, I recognized privileges I had coming from a middle-class household with quality healthcare and education. Although my father is Black, and worked with mostly Black community members, he faced challenges being perceived as an outsider. I experienced this, too, in my work with our community partners, even ones who shared similar racial or gender identities to me. Skin color alone is not enough to ensure I understand another person's experience. I am also not a military veteran. At times, my ability to relate to our targeted community is limited because of our differences in physical ability, health conditions, and living in rural vs. urban areas.

### Our community-engaged implementation science

Community-engaged dissemination and implementation involves collaborating with those impacted by innovations to increase use of innovations (2). We aim to reduce healthcare disparities in implementation by addressing inequitable access to, quality of, or outcomes from innovations, so we believe community engagement in implementation practice and science can be necessary, although not sufficient, to ameliorating inequities.

We spent over two years learning principles of Community-Based Participatory Research (CBPR) to inform community-engaged implementation science and practice (3). In 2020, early in the COVID-19 pandemic, we sought a community organization to partner in research with us. We sought partnerships with veterans of the United States military living in rural Arkansas because (1) we work in the Veterans Health Administration, (2) there are disparities in access to mental health care for rural-dwelling people and (3) veterans in rural areas have higher suicide death rates than those in urban areas (4, 5). In August 2020, we identified a good fit with a non-profit community organization led by veterans living in rural areas. We were accepted into a year-long university course to learn CBPR together. Our community-academic partnership co-designed an intramural university pilot study. We were awarded this grant in March 2022 and it is ongoing. One long-term goal of our partnership is to implement through Arkansas community organizations evidence-based interventions used in healthcare to prevent suicide among veterans (6). We are culturally adapting a suicide prevention intervention with and for rural veterans to be delivered by peers, rather than healthcare providers, to prepare for implementation (7). We are assessing implementation barriers and facilitators to deploying this intervention through community organizations to reach more rural veterans.

### Brief introduction to reflexivity

Reflexivity is the process of engaging in self-reflection about who we are as scientists, how our subjectivities and biases inform the process, and how our worldview is shaped by the research we do and vice versa (8). Learning how to insert reflexivity in the research process early on requires scientists to think critically about the utility, ethics, and value of what, who, and how we study (9). When developing studies, researchers should ask: “*Why do I want to research this group? Who is represented within the research team? What positions of power do I hold and how does that influence my agenda?*”.

Specific to implementation science, practicing reflexivity is essential for health equity and not practicing reflexivity can lead to unnamed power differentials and unexamined harms, even in “successful” implementation efforts (10, 11). There are examples of using “periodic reflections” (12) or tools to capture overlapping power structures within and among people in implementation efforts (13) that could capture important contextual data and function as reflexive practice. Initiating these conversations with researchers and community partners early on allows scientists to acknowledge their positionalities and encourages thoughtful engagement throughout the implementation process (14).

In this perspective on reflexivity, we started as beginners. We knew critical reflection about power was a cornerstone for health equity and community engagement (10, 11). Yet, we had not practiced reflexivity consistently. We thought it might be valuable to others to share how we incorporated reflexivity as beginners, and what we found difficult, feasible, and valuable.

### Methods piloted to practice reflexivity

We practiced reflexivity using five different methods. First, we explicitly identified our positionality in relation to the spaces we inhabit at work. We used the Cultural Identity Inventory (15) independently and discussed our own inventories through many conversations. We named some of our identities and lived experiences. We discussed how the power, privilege, or marginalization of these identities shifted based on the environment—whether we were around others “like us” or minoritized in other situations. Identifying our positionality was a foundational step that we referred to throughout all other methods.

Second, we scheduled time to write fieldnotes every two weeks after our largest meeting with community partners. Fieldnotes are written observations or analysis while “still in the field” conducting research (16). Other interactions with community members were occurring throughout, such as group text messages, one-on-one telephone calls about issues or questions, and small group meetings face-to-face for work intensive tasks in which we needed each other's input (e.g., determining participant inclusion/exclusion criteria of our study). Therefore, our fieldnotes often documented many points of engagement with community members. In consultation with a mentor, we created a template for entries to standardize domains for reflection. Our template prompted us to identity recent events in the work, our feelings and why we felt that way, our actions and whether they were just or helpful, potential next steps and rationale, and resources for learning.

Third, we had periodic, monthly, verbal discussions with two other mentors on technical aspects. Although much of the other introspection and consultation was focused on process, we had logistic and technical questions about forming a community-academic partnership and determining a suitable implementation science study. Examples of logistic questions included: *How should the discussions go when we first meet potential community partners? What parts of a grant can community members contribute to and how should we elicit their ideas given different levels of technology and health literacy?.*

Fourth, we regularly synthesized our “head notes” by debriefing *via* telephone or video after interactions with community members. Head notes were coined in a seminal book by anthropologists as memories, feelings, and instances that live in one's memory, not on paper (as fieldnotes), and thus, symbolize our perspectives based on our lived experiences (16). Head notes capture feelings and physical sensations from memories of interactions, the more affective part of interpersonal interactions with community members or other conduits needing engagement (e.g., information technology specialists, budget managers). Head notes are not always verbally debriefed, but we only used verbal debriefing and did not record these consistently in writing (although they are reflected in many of our fieldnotes, too). As examples, we discussed feelings such as excitement when we connected with community members or figured out a plan forward, and frustration about handling the administrative and scientific load in addition to after-hours meetings when others were absent. We reserved 10–30 min after interactions to share head notes, talk about how it felt, how feelings connected to our positionality, and name tensions or successes. Inevitably, that information funneled into, understanding interactions in context of our positionality in relation to community members and each other.

Fifth, we consulted original reference materials, such as seminal readings (17, 18) and modules from our university's CBPR course. This knowledge building occurred after other reflexive practices because, through those other practices, we realized we needed to “zoom out” and re-read examples of other community-engaged research. We sometimes consulted reference materials with a specific question about a best practice.

## Discussion

### Challenges and solutions to practicing reflexivity in implementation science

We initially invited our community member partners to identify their positionality with us by completing the Cultural Identity Inventory. After many delays, we learned this task was too unfamiliar and time consuming for community members. Therefore, we adapted and agreed to circulate brief biographies about identities and lived experiences that were important to us to the group *via* text message, and encouraged photos or videos to showcase some of these experiences, akin to a less intensive version of photovoice research methodology used with other military veterans (19). For a group that met virtually starting during the COVID-19 pandemic, this created warmth, connection, and understanding of identities with power or marginalization for some.

We planned to write fieldnotes weekly, but quickly learned weekly fieldnotes were difficult, especially because we already engaged in daily writing for other work. For practical reasons, we did this biweekly. Yet, writing fieldnotes is a practice anthropologists maintain regularly, and it would be important to prioritize time and discipline to writing them throughout implementation efforts. One of us sometimes avoided fieldnotes if there had been a difficult interaction due to not wanting to relive the experience, while the other approached fieldnotes to process difficult interactions.

Consulting with other mentors was feasible. Comparing head notes out loud was feasible, perhaps because we were used to speaking frequently for other work tasks. We prioritized by scheduling time in our calendars.

### Value of practicing reflexivity while engaging communities in implementation science

We needed different methods as we moved from naïve to more experienced community-engaged researchers. Different methods served unique functions. Consultation with mentors was less necessary over time, and less focused on power structures, so we decreased the frequency. Identifying our own positions was necessary to name dynamics that typically go unnamed and to understand others' needs and perspectives. After openly discussing our positionality with community members, we had a “shorthand” way to communicate, allowing us to briefly say the lens through which we were experiencing something and have others understand it.

Fieldnotes especially required commitment and were important not to minimize. Written fieldnotes enabled us to reflect on our successes and missteps, funneling into mid-course corrections. This was the only private reflection activity, so it allowed for unfiltered catharsis. In fieldnotes, we sometimes categorized experiences under certain dynamics we were monitoring, which simplified some overwhelm.

Head notes allowed us emotional catharsis, to “fill in gaps” of fieldnotes or consultations with mentors and generate insights from each other's perspectives. Comparing head notes was more important than we thought, because the work was emotionally and cognitively intense—acknowledging unearned power among us and community members and ways it manifested in harmful behavior, or balancing tension between relationships, administration, and CBPR principles such as building trust and equitable compensation. Plus, we were translating CBPR into implementation science, which has some overlaps (20), but involves newer or, perhaps unpublished, realms of community members' engagement in implementation efforts, such as involving them in studying barriers to uptake of innovations (vs. developing innovations) and their role as implementation actors (vs. recruiters or participants). Head notes helped us process the overwhelm and realize how our subjectivities strengthened or threatened the work. In a seminal book on fieldnotes, one anthropologist asserted head notes were more important than fieldnotes (16), although we found those methods to be complementary.

Referencing learning materials reminded us of ideal CBPR principles, and discussion with mentors allowed for learning technical aspects while balancing real-world challenges and CBPR principles. Consultation with mentors and reference materials allowed us to name the broader work of community-engaged research and to find validation in others' missteps or difficulties, hope of moving forward when it seemed difficult, and expertise from others.

### Examples of “outcomes” of practicing reflexivity in our work

When finding a community partner, comparing head notes together made us realize the unease we felt, which prompted us to obtain more mentorship. This led to more explicit conversation with potential community partners about our skills, which helped clarify we were not a good fit for some community organizations and a great fit with others.

Once partnered and deciding on a research topic, identifying our positionality highlighted a tension between areas the community wanted to address and a narrower set of areas we, as academics, had training and skillsets to work on. Because we were aware of our limits and agenda, we named that explicitly, and decided to have several critical conversations as a partnership, then use specific consensus building process to decide on an area we could all work on feasibly and with genuine interest. We acknowledged that the communities' other concerns were not to be tabled forever, and as we nurture our partnership, we can revisit other areas.

In the process of preparing a research study together, we used fieldnotes to reflect on challenges in figuring out a section of the design, unmet deadlines, miscommunications about methods, and it helped us identify unhelpful dynamics. We readjusted explicit norms, altered meeting modalities, and recruited new community members to join our community-academic partnership.

As we have been adapting a suicide prevention intervention through ongoing focus groups, our head notes and fieldnotes help us understand differential power and privilege within the focus group participants. Thus, we purposefully structured our data collection and adaptation processes with best practices for inclusivity. Specifically, we acknowledged community desire to have healthcare professionals as “experts” in focus groups with community members, and how their presence might create silence among community participants because of perceived power of formal training over lived experience. Thus, we invited only community members to meet for the first three focus groups and added healthcare professionals after community members anonymously voted they were ready for healthcare professionals to join. We agreed not to use professional titles and to compensate community member and healthcare professional participants the same amount.

### Advancements and future work

While implementation scientists concern ourselves with acting, “doing,” and impacting, perhaps elevating efficiency above quality, we are challenged by the “sitting” and “being with” community members (and other implementation actors) needed for deep understanding that might lead to the most robust, equitable outcome (21). Implementation involves highly complex and connected networks of people, bringing their own perspectives and differing experiences with power and marginalization, which affect the work (22, 23). Practicing reflexivity, although maybe not as expertly as an ethnographer or anthropologist, is a useful approach to community-centered, impactful implementation science and practice.

As we consider necessary skills for dissemination and implementation science and practice committed to health equity (24), reflexivity might be considered a core element based on fields of anthropology, sociology, and others deeply embedded in understanding communities and knowledge production (11). It would be interesting to observe which actionable methods to practice reflexivity other implementation scientists or practitioners find useful, and report these in publications.

There are existing resources for reflexivity in implementation science. Within the field, our anthropology colleagues and those trained in ethnography can be helpful consultants to guide these processes in the work. There is guidance on reflexivity in quantitative methods (14). Other suggestions may be found using periodic reflections (12), questions suggested by others on power in implementation science (10, 23), or in the journal, *Reflective Practice: International and Multidisciplinary Perspectives*.

Practicing reflexivity, we began to become aware of power imbalances, named tensions, and minimized power dynamics between us and community members, and built practices for inclusivity and effective work toward health equity. We encourage implementation scientists, especially those working for or with communities, to experiment with reflexivity practice.

## Data Availability

The original contributions presented in the study are included in the article/Supplementary Material, further inquiries can be directed to the corresponding author.
